# Coarse sea spray inhibits lightning

**DOI:** 10.1038/s41467-022-31714-5

**Published:** 2022-08-02

**Authors:** Zengxin Pan, Feiyue Mao, Daniel Rosenfeld, Yannian Zhu, Lin Zang, Xin Lu, Joel A. Thornton, Robert H. Holzworth, Jianhua Yin, Avichay Efraim, Wei Gong

**Affiliations:** 1 State Key Laboratory of Information Engineering in Surveying, Mapping, and Remote Sensing, Wuhan University, 430079 Wuhan, China; 2 Institute of Earth Sciences, The Hebrew University of Jerusalem, Jerusalem, 91904 Israel; 3 School of Remote Sensing and Information Engineering, Wuhan University, 430079 Wuhan, China; 4 School of Atmospheric Sciences, Nanjing University, 210023 Nanjing, China; 5 Joint International Research Laboratory of Atmospheric and Earth System Sciences & Institute for Climate and Global Change Research, Nanjing University, 210023 Nanjing, China; 6 School of Electronic Information, Wuhan University, 430079 Wuhan, China; 7 Department of Atmospheric Sciences, University of Washington, Seattle, WA USA; 8 Department of Earth and Space Sciences, University of Washington, Seattle, WA USA

**Keywords:** Atmospheric dynamics, Atmospheric chemistry

## Abstract

The known effects of thermodynamics and aerosols can well explain the thunderstorm activity over land, but fail over oceans. Here, tracking the full lifecycle of tropical deep convective cloud clusters shows that adding fine aerosols significantly increases the lightning density for a given rainfall amount over both ocean and land. In contrast, adding coarse sea salt (dry radius > 1 μm), known as sea spray, weakens the cloud vigor and lightning by producing fewer but larger cloud drops, which accelerate warm rain at the expense of mixed-phase precipitation. Adding coarse sea spray can reduce the lightning by 90% regardless of fine aerosol loading. These findings reconcile long outstanding questions about the differences between continental and marine thunderstorms, and help to understand lightning and underlying aerosol-cloud-precipitation interaction mechanisms and their climatic effects.

## Abstract (web summary)

Previous hypotheses cannot fully explain the large lightning excess over land compared to ocean. It is found that coarse sea spray that create large drops precipitates cloud water before it can freeze, thus robbing the fuel for cloud electrification

## Introduction

The electrification of deep convective clouds (DCC) occurs when ice crystals collide with graupel in supercooled water clouds with a differential transfer of electrical charge upon collision. The crystals, which tend to accumulate a net positive charge, rise with the updraft while the graupel, which tend to accumulate a net negative charge and descend gravitationally, thereby building the electric field that leads to lightning discharges^1,2^. Therefore, more electrification occurs in clouds with stronger updrafts and more supercooled liquid water content (SLWC)^1–3^. The updraft intensity is dominated by the convective available potential energy (CAPE), but it can be invigorated further by adding the fine aerosols (dry radius < 1 μm), which act as cloud condensation nuclei (CCN)^4,5^. The added CCN nucleate more numerous and smaller cloud droplets, which are slower to coalesce into raindrops, thus surviving longer in the updraft and reaching greater heights where it enriches the SLWC^6,7^. Moreover, additional nucleated cloud droplets enhance the condensation efficiency, leading to the additional release of latent heat, thus accelerating the updrafts^8^. These considerations explain why lightning frequency has been observed to increase with both CAPE and CCN, but decrease with greater distance between cloud base height and the freezing level, over both tropical land and ocean^9–12^.

However, the average lightning frequency for the same rainfall amount is generally far smaller over tropical ocean compared to land^13^ despite similar meteorology. An outstanding question in this regard is to what extent is this land-ocean contrast explained by the lower cloud base heights and updrafts over ocean^14,15^ and/or the differences in CCN concentrations^6^. The known differences cannot explain why the product of CAPE and precipitation amount correlates well with the lightning frequency over land, but not at all over ocean^16^, and leaves much of the variability of oceanic lightning unexplained. Previous studies showed that added aerosols nucleate more cloud droplets which leads to convective invigoration and electrification. Other favorable meteorology conditions also contribute to the development of convection with strong updraft, such as high precipitable water (PW) and low wind shear^17,18^. Additionally, ultrafine aerosol particles (UAPs) could invigorate the DCC by secondary activation with the resultant added latent heat of condensation^8,19^. This hypothesis of UAP invigoration needs to be further verified based on observations. However, as we show here, coarse sea salt aerosols with a dry radius > 1 μm (CSS, or sea spray) have the opposite effect of small CCN, i.e., the CSS nucleate fewer but larger cloud drops which coalesce to raindrops and precipitate faster^20–22^, thus leading to weakened DCC.

Here, we test the hypothesis that fine and coarse marine aerosols have opposite effects on lightning frequency using properties of large tropical DCC clusters and lightning after isolating the effects of meteorology. The DCC properties used here are cloud top temperature (CTT), lifetime, rainfall, and lightning observed over tropical land and ocean – Africa and the adjacent oceans, respectively, between 50°W to 50°E and 20°S to 20°N during 5 years. The aerosol concentrations are obtained from the Modern-Era Retrospective Analysis for Research and Application Version 2 (MERRA-2) reanalysis data, and lightning stroke densities are from the World Wide Lightning Location Network (WWLLN).

## Results

### Land-ocean contrasts of lightning

**Fig. 1:**
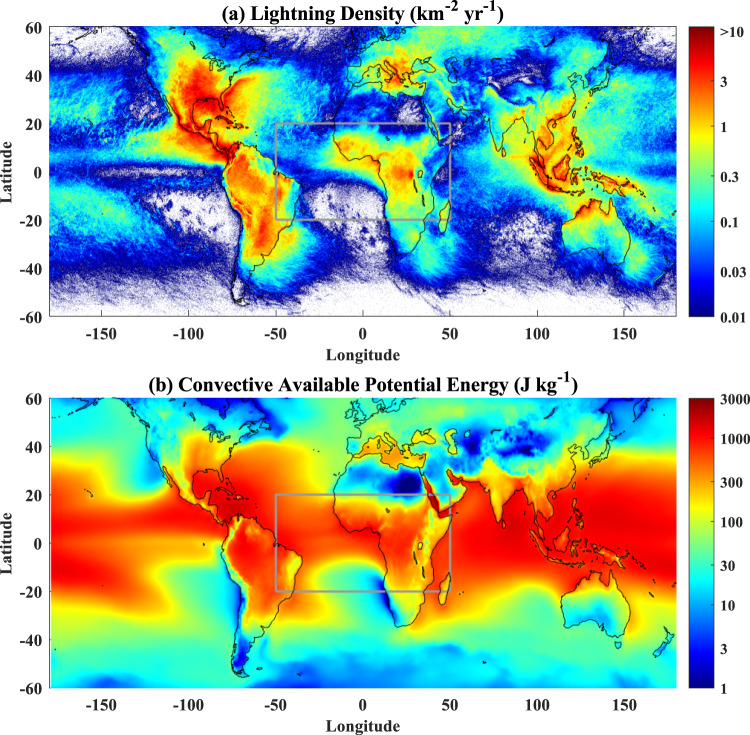
Global distribution of lightning density and convective available potential energy (CAPE). The lightning density (**a**) and CAPE (**b**) are generated from World Wide Lightning Location Network (WWLLN) and National Centers for Environmental Prediction (NCEP) Final Operational Global Analysis data from 2013 to 2017. The gray rectangle indicates the region of interest from 50°W to 50°E and 20°S to 20°N.

**Fig. 2:**
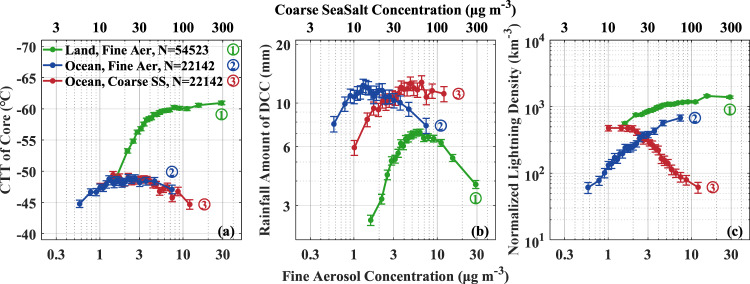
Relationships between aerosols and deep concective cloud (DCC) properties. The dependence of the cloud top temperatures (CTTs) of convective core (**a**), domain-averaged rainfall amount (i.e., rainfall depth) throughout DCC lifetime (**b**), and lightning flashes per km^3^ of rainfall (**c**) are given as a function of fine aerosol mass concentrations (bottom abscissa scale) over land (green lines) and ocean (blue lines). The red lines give the dependence on coarse sea salt (CSS) aerosol mass concentrations over ocean (top abscissa scale). The I-type vertical bars indicate standard error. The aerosol bins are at intervals of 5% of the cases. The total number of data points for each line is shown in the legend.

Fig. 1a shows that lightning occurs mostly over land compared to ocean. Lightning frequency has been shown to significantly increase with CAPE^16^, which dominates the updraft intensity. However, the CAPE over land and ocean is similar or even larger over ocean in some areas (Fig. 1b). The CAPE cannot explain the significant land-ocean contrast of lightning. The relationships of fine aerosols with CTT of convective core and rainfall amount (green and blue lines for over land and ocean, respectively, in Fig. 2a, b) show the convective invigoration as we previously documented in the study of Pan et al.^18^. Fine aerosol invigoration for CTT over land is much stronger than that over ocean with the change in CTT around −12 °C from clean to polluted conditions, while the corresponding change over ocean is around −4 °C. Also, the rainfall amount is almost tripled from clean to optimal fine aerosol concentration (5 μg m^−3^) over land, but increases by only a factor of 1.6 over ocean (Fig. 2b). The results are robust to partitioning the data into three bins of CAPE and further dividing each of them into three bins of PW (Supplementary Figs. 1, 2, and Table 1).

Despite the much weaker invigoration of CTT and rainfall per fine aerosol increase over ocean, the lightning density over ocean increases much more than over land. Lightning density increases by a factor of ×11 from low to high mass concentrations of fine aerosols over ocean, compared to only ×2.6 over land for a given rainfall amount. The results are also robust to partition into CAPE and PW bins (Supplementary Figs. 3 and 4). In stark contrast to the fine aerosol ×11 effect on increasing lighting, we find lightning reduced down to ×0.1 associated with increased CSS concentrations. This large decrease was associated with an increase in warm rain with a moderate warming of CTT (mainly at low PW conditions). This indicates the effect of CSS on enhancing warm rain at the expense of cloud water reaching the supercooled levels, which incurs weakening of the mixed-phase precipitation and lightning.

Although the CSS is indicated to weaken the convection and lightning, it lengthens the lifetime of convection (Supplementary Figs. 5 and 6). However, with the CSS enhancement of warm rain, a convective cell with faster precipitation forming processes is expected to live a shorter time. This apparent contradiction may be explained by the fact that the entities are multicell systems. A weaker convection over ocean consumes the CAPE more slowly with the increase in CSS, thus lengthening the lifetime of the whole convective cluster. Additionally, to further ensure the effects of meteorology on aerosol-driven lightning variation. Other relevant meteorological factors are examined referring to the previous studies^18,23^, including 450 hPa vertical motion, surface and 450 hPa relative humidity, and 850–200 hPa wind shear. The results show that the aerosols effects are robust and independent on meteorology over both land and ocean (Supplementary Fig. 7). Furthermore, surface wind speed affects CSS^24^, but there is no indication to any dependence on wind speed of the effects of a given CSS on lightning (Supplementary Figs. 9 and 10).

### Fine and coarse aerosol effects contrast

**Fig. 3:**
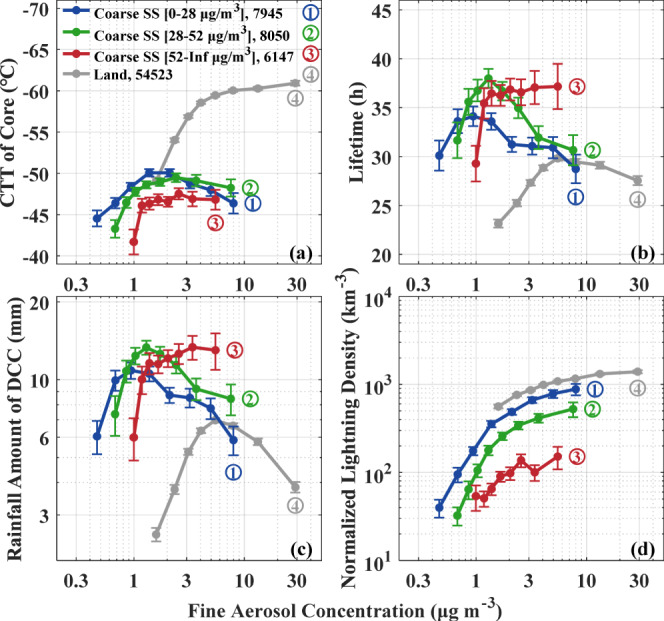
Relationship between deep concective cloud (DCC) properties and fine aerosols for three fixed bins of coarse sea salt (CSS) aerosols over ocean. The DCC properties are: **a** cloud top temperature (CTT) of core, **b** lifetime, **c** rainfall amount, and **d** normalized lightning density by rainfall amount throughout the DCC lifetime. The gray lines are for DCC over land, replicating the green lines of Fig. 2. The I-type vertical bars indicate standard error. The same percentile of 5%, 15%, 30%, 50%, 70%, 85%, 95%, 100% for fine aerosol bins are used over ocean, respectively.

The fine aerosol concentrations are positively correlated with the CSS aerosol concentrations over ocean, thus the effects of these two aerosol types compete with each other. To separate the effects, Fig. 3 shows the relationships of DCC properties with fine aerosols, partitioned for the lower, middle and upper thirds of the CSS aerosols. According to Fig. 3, adding fine aerosols while holding CSS at low concentrations leads to a substantial convective invigoration over ocean, as manifested by a −6 °C cooling of CTT (Fig. 3a) and a factor of ×22 enhancement in lightning (Fig. 3d)—much larger than the ×2.6 over land. According to Fig. 3d, the dependence of lightning on fine aerosols over land is very similar to that over ocean with low CSS (blue and gray lines), except for a 25% downward shift of the oceanic lightning frequency for the same fine aerosols. This 25% difference may be ascribed to the thermodynamic differences between land and ocean^12^.

The lightning sensitivity is very high for low concentrations of fine aerosols and tends to saturate at high aerosol concentrations. The fine aerosol concentration span between the 5% to 95% percentiles over ocean (0.5–7.5 μg cm^−3^) is 1/3 compared to over land (1.5–30 μg cm^−3^). Therefore, the oceanic clouds occur mostly at the steep part of the curve, whereas continental lightning is in the more saturated part of the curve. These differences in aerosol concentrations and sensitivities explain much of the lower occurrence of lightning over ocean compared to land. The results also explain the greater apparent sensitivity of oceanic lightning to aerosol input compared to over land. This is evident by the much larger lightning enhancement over ocean compared to land from the low to high end of fine aerosol concentrations, whereas the CAPE is similar for both land and ocean. These changes are consistent with convective invigoration, and with a large increase of mixed-phase precipitation at the expense of warm rain, especially over ocean.

Given this large sensitivity to fine aerosols, marine clouds with relatively large concentrations of fine aerosols produce almost as much lightning as continental clouds with the same fine aerosols. However, when increasing the CSS in such clouds from the lowest to the highest third of CSS concentrations causes a warming in CTT (Fig. 3a) and a decrease in lightning (Fig. 3d). The decrease in lightning is greatest (×0.2) for the largest amount of fine aerosols, because the CSS can restore more coalescence which would otherwise be suppressed by the higher concentrations of fine aerosols. The CSS suppression of lightning is also supported by the variation of ice water path (IWP) of convective precipitation (Supplementary Fig. 11). IWP significantly increases with fine aerosol under a fixed CSS (Supplementary Fig. 11a), especially under low CSS concentration. The CSS decreases the IWP at a comparable magnitude as fine aerosols increase it. The CSS inhibition of IWP is strongest with the largest fine aerosol concentration. It appears as if CSS counteracts the mixed-phase precipitation enhanced by fine aerosol through enhancing warm rain, which is consistent with the observed aerosol-driven lightning variations.

The red line in Fig. 2c indicates a possible decrease of ×0.1 when changing from the lowest to the highest 5% bins of the CSS concentrations. At the same time, adding CSS restores the warm rain process, and increases lifetime and rainfall amount (Supplementary Fig. 5). These changes are consistent with convective weakening and a considerable increase of warm rain at the expense of mixed-phase precipitation. Additionally, raindrops initiated by the sea spray can grow by collecting small cloud droplets that form on the fine particles, thereby cleansing the air over ocean^25^. Thus, without CSS, marine air mass would have been much more polluted than actually observed. This indicates that CSS may also inhibit the fine aerosol convective invigoration through the cleansing process driven by the enhanced warm rain, contributing to the much lower lightning density over ocean.

## Discussion

**Fig. 4:**
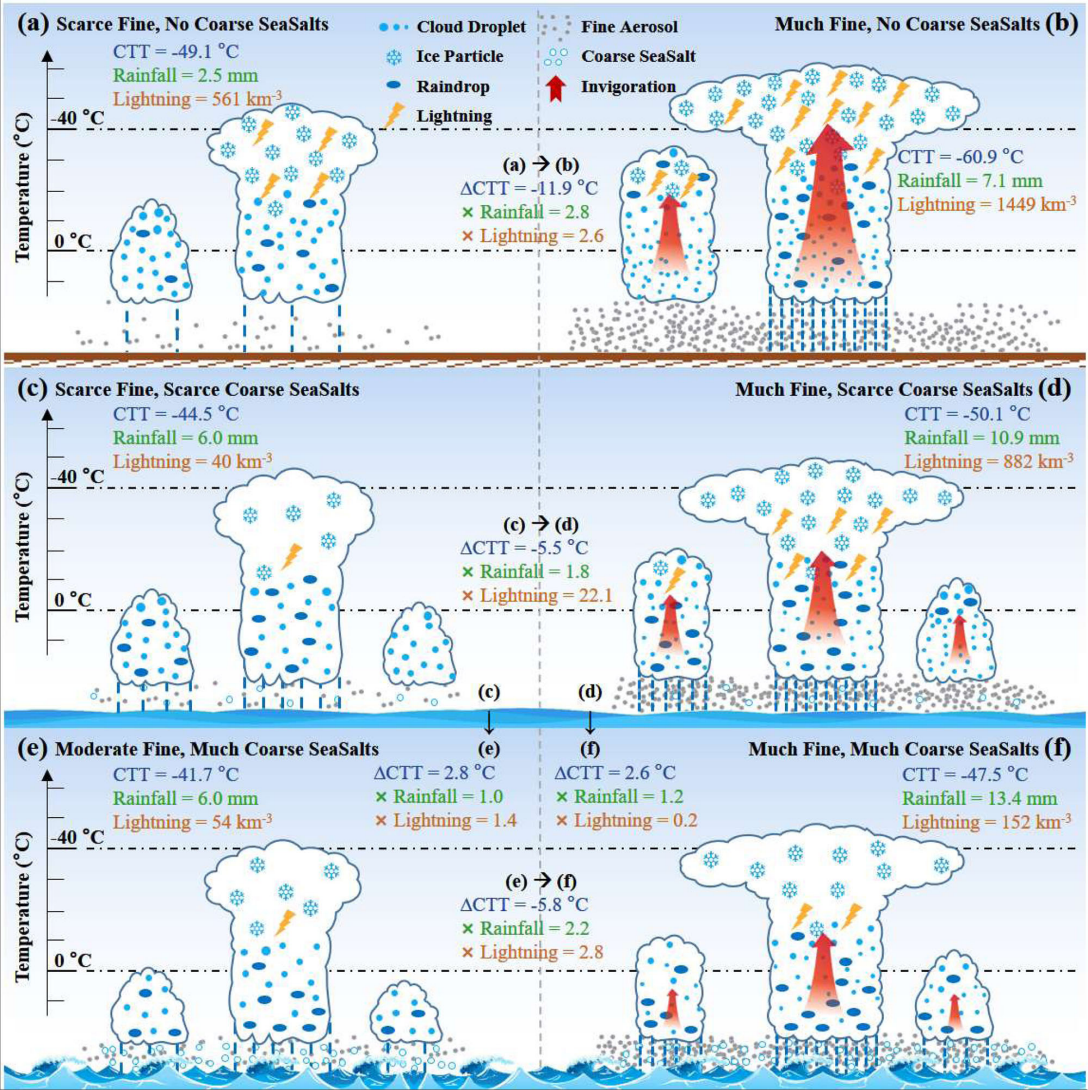
Schematic illustration of the aerosol effects on DCC properties and lightning over land and ocean. The convective core cloud top temperature (CTT), domain averaged rainfall amount (i.e., rainfall depth) throughout deep concective cloud (DCC) lifetime, and lightning flashes per km^3^ of rain volume over land (**a**, **b**), ocean with the lowest third (blue lines in Fig. 3) of the coarse sea salt aerosols (**c**, **d**) and ocean with the upper third (red lines in Fig. 3) of the coarse sea salt aerosols (**e**, **f**), respectively. The left column (**a**, **c**, **e**) indicates the conditions at the smallest 5% bin of fine aerosol mass concentrations, and the right column (**b**, **d**, **f**) indicates the conditions at the largest 5% bin. The temperature scale marks the isotherms from 0 °C to −70 °C every 10 °C. The differences in cloud properties and lightning between panels are posted under the arrows connecting the compared panels. The ∆CTT indicates the change in CTT of the core from clean to optimal fine aerosol concentrations. The × rainfall indicates the respective enhancement factor of rainfall amount. The × lightning indicates the change of normalized lightning frequency from clean to maximum fine aerosol concentrations, as lightning keeps increasing with more fine aerosols beyond the optimal aerosol concentrations for rainfall amounts. The red updraft arrows symbolize the added invigoration due to adding fine aerosols over land (**a**, **b**), ocean with few (**c**, **d**), or with much coarse aerosols (**e**, **f**), respectively.

Previously, known differences in thermodynamics and aerosols could not explain much of the large excess in lightning activity over land compared to ocean for the same rainfall amount. Herein, we resolved this question by quantifying the effects of both fine aerosols and coarse sea salt aerosols (CSS) on tropical DCC properties and lightning over ocean compared to over land. The results are based on the WWLLN measurements. Other independent lightning measurements with published characterization of their sensitivity at the time-space domain of this study were not available for an independent replication of these results. The gained insights, as summarized and illustrated in Fig. 4, are:

DCC over land have only slightly more lightning than DCC over ocean with the same fine aerosol concentrations and scarce CSS (Fig. 3d). This finding is somewhat surprising given the weaker DCC invigoration over ocean, which means smaller enhancement of graupel and ice crystal concentrations that their collisions electrify the clouds. The lesser invigoration of oceanic clouds is indicated by the warmer and lower cooling of CTT and the smaller rainfall enhancement with added fine aerosols (Fig. 4).

Under conditions of scarce CSS, the lightning enhancement from smallest to largest fine aerosol concentrations over ocean (×22) is much greater than over land (×2.6), as shown in Fig. 4a, d. The lightning sensitivity to fine aerosols is largest at the fine aerosol concentrations < 1.5 μg m^−3^, which exist over ocean but are mostly absent over land. On the other end, the effect tends to saturate at the heavy fine aerosol over land. However, under conditions of heavy CSS concentrations, added fine aerosol concentrations from smallest to largest fine aerosol concentrations over ocean only cause slight invigoration and lightning enhancement (×2.8), which is a much lesser extent than under scarce CSS (Fig. 4e, f).

Increasing CSS causes DCC weakening, which is the opposite of fine aerosol DCC invigoration. This is manifested in warming CTT and a large decrease in lightning frequency for the same fine aerosol concentrations. The decrease is strongest (around ×0.2) for the largest fine aerosol concentrations (Fig. 4d, f), where the CSS restores most strongly coalescence and warm rain on the background of the most suppressed coalescence by fine aerosols.

In summary, fine and coarse soluble aerosols have opposite effects on convective invigoration and electrification. Although it is already well documented that fine aerosols suppress coalescence and invigorate DCC and their electrification, it is the first time that coarse sea spray aerosols are documented to weaken DCC and suppress lightning. In contrast, coarse continental aerosols in the form of desert dust were reported to increase lightning over land, although to a lesser extent than fine aerosols^10^. The probable mechanism of the coarse sea salt aerosols is by enhancing drop coalescence and replacing mixed-phase precipitation with warm rain, thus “killing” cloud electrification.

These findings have climatic importance. CSS-induced conversion of mixed-phase to warm rain means less release of latent heat of freezing, thus less upward transport of heat for the same surface rainfall amount^26^. This requires more rainfall to satisfy the radiative deficit of the troposphere over ocean. Therefore, accounting more correctly for the contrasting effects of fine and coarse marine aerosol on DCC would improve the calculated rainfall amounts and vertical distribution of latent heating, which drives much of the atmospheric circulation system.

## Methods

### Data collocation

The region of interest is from −50° W to 50° E and −20° S to 20° N, containing tropical Africa and central Atlantic. We focus on the tropical region to minimize the effects of meteorological disturbances and maximize the role of DCC in producing precipitation. The data used in this study were obtained from multiple observations for cloud, precipitation, aerosol and meteorology from January 2013 to December 2017. The Meteosat Second Generation (MSG) geostationary satellite is used to identify the cloud properties, including cloud phase and cloud top temperature (CTT). Rainfall data is acquired from Integrated Multi-satellitE Retrievals for Global precipitation mission (IMERG) product of the Global Precipitation Measurement (GPM) mission. Environmental data is acquired from National Centers for Environmental Prediction (NCEP) Final Operational Global Analysis data. Additionally, the GPM Dual-frequency Precipitation Radar observations are used to analyze the aerosol-driven IWP variations for convective precipitation (Supplementary Fig. 11). The IWP is calculated based on the radar reflectivity factor, referring to the previous studies^3,27,28^.

Aerosol conditions at the boundary layer were obtained from the MERRA-2 reanalysis products. MERRA-2 has assimilated the multiple satellites (such as Moderate Resolution Imaging Spectroradiometer) and Aerosol Robotic Network (AERONET) observations^24,29^. Based on the long-term comparison in global, the MERRA-2 and AERONET aerosol optical depth generally has a good agreement with an average correlation of 0.85, and this agreement even reaches to 0.93 for aerosol over ocean^24^. The specific information of all the cloud, aerosol, precipitation and meteorological products and variables used in this study are the same as in the study of Pan et al.^18^ and fully described there.

The WWLLN is the longest-running global lightning network, with coverage beginning in August 2004. WWLLN locates lightning using dozens of radio receivers in the very-low-frequency range (VLF; 3–30 kHz) located throughout the world. WWLLN detects the majority of all lightning-producing storms with the accuracy of lightning locations about 5 km and high-temporal resolution^30,31^, even in regions with no stations within 2000 km^32,33^. Holzworth et al.^34^ showed that the WWLLN detects about 70–80% of all strokes above 40 kA compared to the New Zealand Lightning Detection Network. This indicates that WWLLN was locating nearly all synoptic thunderstorms^32^. The relative detection efficiency for lightning is near 100% over both of land and ocean consistently, except over the ice of Greenland and Antarctica with rare lightning^31^. Actually, the lightning energy distribution over land and ocean are statistically similar for the studied DCC (Supplementary Fig. 12). This supports a consistent detection efficiency for the total lightning over both land and ocean.

The observed magnitude of the lightning enhancement over the shipping lane was similar in both WWLLN and LIS (Tropical Measuring Mission Lightning Detecting Sensor). The LIS signal was noisier, probably due to the smaller sample size from the low and relatively narrow swath of orbit^11^. Since LIS is an optical sensor from low orbit, it tends to detect most efficiently the weak lightning that occurs near cloud tops, whereas WWLLN uses a very-low radio frequency. Virts et al.^35^ showed that the sensitivity of WWLLN compared to LIS is somewhat greater over the oceans than over land^35^. A detailed discussion about the performance of WWLLN data is provided in Supplement Information.

The analysis in this paper is based on the lightning detected from 2013 to 2017. All data are matched at the spatial resolution of the MSG (9 km × 9 km) based on the nearest method in space. Further, considering the availability of GPM IMERG for DCC, we match all data at 30 min intervals (IMERG time intervals) based on the linear interpolation method in time.

### Tracking convective systems properties throughout their lifecycle

We use a fixed window of 10° × 5° in longitude and latitude to capture the full lifecycle of convective systems, while moving mostly with a dominant zonal component. The specific method of defining and capturing a convective system throughout its whole lifetime is provided in the study of Pan et al.^18^. A convective core is defined as its area occupied by IMERG rainfall rate > 1 mm/h with a cloud with an ice phase top. Each case of DCC is constituted of a convective core and its ice anvil. The DCC lifecycle begins with the initiation of a convective core to the dissipation of its related anvil. Here, a tracked convective event indicates the whole evolution of a multicellular convective system at a fixed domain (10° × 5° in longitude and latitude). The multicellular systems in the same area interact and form cloud clusters, which are our objects of interest. Moreover, all convective clouds at the same window have similar interactions with aerosols due to similar environmental conditions.

The domain-averaged rainfall amount of DCC with the unit of mm is the integrated rainfall rate throughout the DCC lifecycle, divided by the total area at a fixed window of 10° × 5°. The normalized lightning frequency for a captured DCC is defined as the total number throughout its lifetime at a fixed window of 10° × 5° per km^3^ of integrated rainfall volume. Based on the method of Pan et al.^18^, 76,665 convective cases with lightning were obtained from January 2013 to December 2017, which are partitioned to 54,523 and 22,142 over land and ocean, respectively. It is noted that 198 and 2439 cases are without lightning over land and ocean, respectively.

## Supplementary information

Supplementary Information

## Data Availability

The dataset containing all the relevant properties of all captured deep convection cases is available at 10.6084/m9.figshare.20098103.v1.

## Code availability

The computer codes used to analyze the data are available from the corresponding author on request.
