# Supplementary material for: Coarse sea spray inhibits lightning
Source: Nat Commun. 2022 Aug 2;13:4289. doi: 10.1038/s41467-022-31714-5 (PMC9345860; doi:10.1038/s41467-022-31714-5)
Supplement: Supplementary file 1 — Supplementary Information [file 41467_2022_31714_MOESM1_ESM.pdf]

Supplementary Information for
Coarse Sea Spray Inhibits Lightning

Zengxin Pan[1,2]†, Feiyue Mao[1,3]†, Daniel Rosenfeld[2,1]*, Yannian Zhu[4,5]*, Lin Zang[6], Xin Lu[1], Joel A. Thornton[7], Robert H. Holzworth[8], Jianhua Yin[3], Avichay Efraim[2], Wei Gong[1,6]*

*Corresponding authors: Daniel Rosenfeld (daniel.rosenfeld@huji.ac.il); Yannian Zhu (yannianzhu@gmail.com); Wei Gong (weigong@whu.edu.cn). †These authors contributed equally to this work.

This PDF file includes:

Supplementary Discussion 1: Isolating the effect of meteorology and aerosols on lightning
Supplementary Discussion 2: Discussion about WWLLN lightning data
Figs. S1 to S11
Tables S1
Supplementary References

Supplementary Discussion 1: Isolating the effect of meteorology and aerosols on lightning

Effect of meteorology on aerosol-driven lightning variation. To further isolate the effects of meteorology and aerosols on lightning, other relevant meteorological factors are examined referring to the previous study[1, 2], including 450 hPa vertical velocity (ω), relative humidity (RH) and 850–200 hPa wind shear (WSH). The results show that lightning significantly increases with precipitable water (PW), convective available potential energy (CAPE) and 450 hPa updraft (blue to red lines), while lightning decrease with the increase in 850–200 hPa wind shear (Supplementary Fig. 7). These are consistent with our previous study[2], which indicates that convection becomes stronger (higher cloud top, longer lifetime and more rainfall amount) under the condition of higher PW, CAPE and updraft, but lower wind shear.

The lightning increase with higher surface RH over land (R=0.34), while it is opposite over ocean, but with very low correlation (R=-0.11). The low correlation over ocean is possibly attributed to the abundant and homogeneous water vapor (RH>70%) at surface over ocean (Supplementary Fig. 8a), resulting in the effect of surface RH is not highlighted. However, the consistently positive relationships are found between lightning and RH at the middle troposphere (450 hPa), where RH distribution is similar over both land and ocean. Therefore, aerosol-driven lightning variations for fine aerosol and coarse sea salt are consistent and significant for whatever meteorology bins (Supplementary Figs. 1 to 7). This indicates that the relationships between aerosols and lightning are general and independent on meteorology.

Isolating the effect of wind speed and coarse sea salt. Additionally, the coarse sea salt concentration is directly driven by the near-surface wind speed over ocean[3]. Here, there is a significantly positive correlation between coarse sea salt and wind speed (R=0.76), but not for fine aerosol (Supplementary Figs. 9a and 9d). Results show that lightning becomes less frequent under the condition of high wind speed (coarse sea salt) and low fine aerosol loading. Moreover, the lightning becomes minimally frequent with the increase both of coarse sea salt and wind speed (Supplementary Figs. 9e and 9f).

To isolate the mixed effects of fine aerosol and coarse sea salt on lightning, we further discuss the aerosol-driven lightning variations with fine aerosol (coarse sea salt) at fixed other aerosol and wind speed. With the increase of fine aerosol from clean to polluted condition, the lightning density for unit rainfall amount increase by an average of one order of magnitude

(Supplementary Figs. 10a-10c). This is independent on coarse sea salt and wind speed, except for some change in magnitude. Additionally, after isolating the disturbance of fine aerosol and wind speed, there is still significant inhibition on lightning with additional coarse sea salt.

In general, the lightning significantly increases with fine aerosol, especially over ocean. However, a comparable significant decrease in lightning is driven by coarse sea salt by enhancing warm rain over ocean. These aerosol-driven lightning variations are consistent and significant for whatever meteorology bins. This indicates that the relationships between two aerosols and lightning are general, comparable and independent.

Supplementary Discussion 2: The suitability of WWLLN lightning data for the study objectives

WWLLN stations and their detection range. The WWLLN is the longest-running global lightning network, with coverage beginning in August 2004. It has about 65 global sensors operating at any time, out of over 80 which are deployed around the world. A plot about the global sensor locations of WWLLN is published every 10 minutes, and shown in http://wwlln.net.

WWLLN locates lightning using the received lightning very low frequency (VLF; 3–30 kHz) sferic as it propagates around the world. As shown in Rodger, et al. [4], stations typically detect sferics out to about 8000 km in the day and over 12,000 km at night. Since ¼ way around the world is 10,000 km, it means that stations within the entire hemisphere (±10,000 km in all directions) centered on the stroke have a good chance to detect that stroke. WWLLN requires that at least 5 stations have good reception of the sferic and currently WWLLN averages about 7 stations participating in every stroke location, globally. Additionally, the strong strokes are often detected by 15 to 20 WWLLN stations (max ever 35 stations).

Tracking lightning evolution with WWLLN data. NASA's satellite lightning detectors use optical transients to detect lightning, such as the Tropical Rainfall Measuring Mission/Lightning Imaging Sensor (TRMM/LIS), International Space Station/Lightning Imaging Sensor (ISS/LIS) and the Geostationary Operational Environmental Satellite/Geostationary Lightning Mapper (GOES16/17 GLM). Thus, the light must exit the top of the cloud with enough intensity to be detected from space. This causes these detectors regularly miss a significant number of cloud-to-

ground strokes, including. Because much of these light exits below the cloud and propagates out parallel to the surface, and is missed by the satellite.

Furthermore, the actual total strokes located in the region of interest are seen only by the low earth orbiters, such as TRMM/LIS and ISS/LIS, but not by the GOES16/17 satellites. However, the actual total strokes for DCC detected by the low earth orbiters are underestimated by 1 or 2 orders of magnitude because of the low temporal resolution of these low earth orbiters [5]. Additionally, the ground-based lightning networks, such as America National Lightning Detection Network (NLDN), EUCLID (European Cooperation for LIghtning Detection network) or NZLDN (New Zealand Lightning Detection Network), require sensors to be ≤ 300 km from a lightning. These networks mainly focus on the lightning over land and coast, but miss the lightning over remote ocean. In contrast, WWLLN is able to detect the lightning continuously throughout the whole convection lifecycle with the real-time observations globally.

Detection efficiency of WWLLN. The relative detection efficiency (RDE) is a measure of how well a given location in the network is being observed relative to the best region in the network. The global RDF of WWLLN is published every day (http://wwlln.net/deMaps). In general, the RDF for strokes is near 100% everywhere, with the lowest relative detection efficiency down to 75% (i.e., Antarctica and parts of Africa and eastern Asia)[6]. Additionally, lightning sferics propagate with very low attenuation over the oceans, and thus lightning is detected over water even better than over land [5]. Note that there is scarce global 'ground truth' data set against which the absolute detection efficiency of WWLLN can be determined presently, especially over ocean.

The latest compilation of WWLLN absolute detection efficiency is given by presented in study of Holzworth, et al. [7] by using a short-range system (New Zealand Lightning Detection Network) as ground-truth. WWLLN detects about 70-80% of all strokes above 40 kA (dominated by cloud-to-ground strokes) and 20% of strokes at 15 kA (dominated by in-cloud strokes). A cloud-to-ground (CG) stroke is typically accompanied with 4 to 6 small in-cloud (IC) strokes. Therefore, about half the strokes WWLLN locates are CGs and about half are ICs.

Also, the WWLLN is more efficient at locating the stronger lightning strokes. Hutchins, et al. [6] showed that the distribution of stroke energies is log-normal within 1 hour, meaning that there is a normal distribution of energies, which would include some large strokes and some smaller ones. Therefore, for a weak storm with just one small stroke, WWLLN may not have located it.

However, for a large number of strokes (i.e. strong storm), WWLLN is likely to have identified strokes from every thunderstorm. Actually, WWLLN was locating nearly all synoptic thunderstorms in 2006, as shown in the study of Jacobson, et al. [8]. Additionally, Supplementary Fig. 12 shows the normalized probability distributions for energy per lightning of tracked DCCs in this study. It shows that lightning intensity distribution over land and ocean are statistically similar. Therefore, the similar lightning intensity distributions support a consistent detection efficiency for the total lightning over both land and ocean.

Possible influence of lightning data on the results. Holzworth, et al. [7] show that WWLLN detects 20% of the strokes at 15 kA, and this ratio is about 70-80% for all strokes. Previous works prove that WWLLN has better detection on lightning over ocean compared to ground-based and TRMM/LIS observations [5, 7]. This indicates that WWLLN was locating nearly all synoptic thunderstorms [8], while it is possibly underestimating the weak strokes, especially over land. Moreover, there is still consistent land-ocean contrast of lightning density based on WWLLN.

Therefore, WWLLN lightning data support our conclusion about the effects of fine and coarse sea salt aerosols over ocean, and the cause of land-ocean contrast of lightning. Actually, the aerosol effect on lightning measured by WWLLN has been compared to that measured by the completely independent TRMM LIS satellite optical sensor over the shipping lanes and found a similar response to the ship emissions [9]. In summary, the results we present in this paper are not likely to be dominated by WWLLN biases.

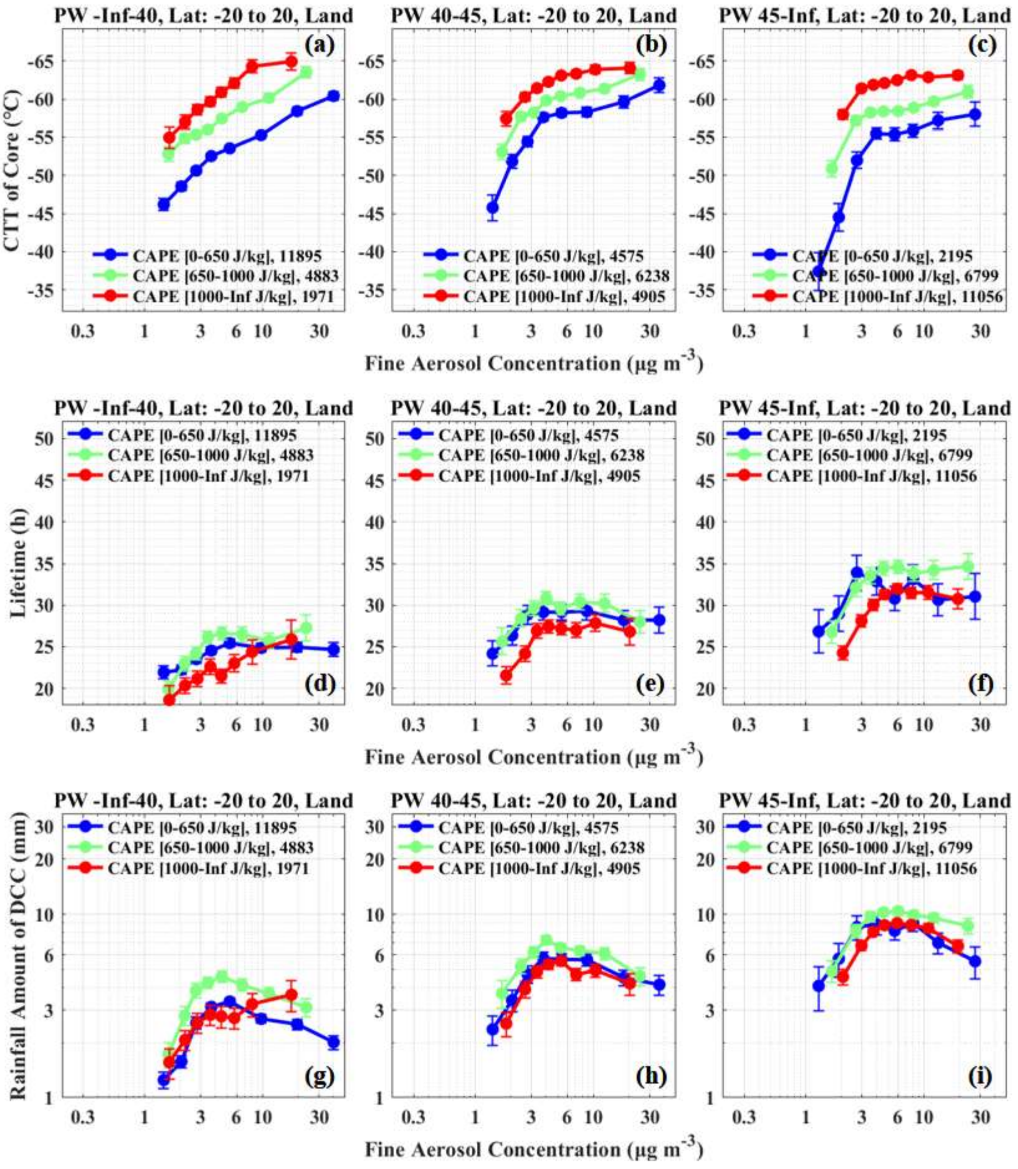

Supplementary Fig. 1 | Aerosol-driven deep concective cloud (DCC) variations with fine aerosol at surface over land. (a-c) cloud top temperature (CTT) of core, (d-f) lifetime and (g-i) rainfall amount of DCC variations are showed under different fixed intervals of precipitable water (PW) over land, respectively. Three lines with different colors (blue, green and red) indicate the low, moderate and high convective available potential energy (CAPE). The I-type vertical bars indicate standard error. The same percentile of 5%, 15%, 30%, 50%, 70%, 85%, 95%, 100% for fine aerosol bins are used over land, respectively. The total number of data points for each line is shown in the legend.

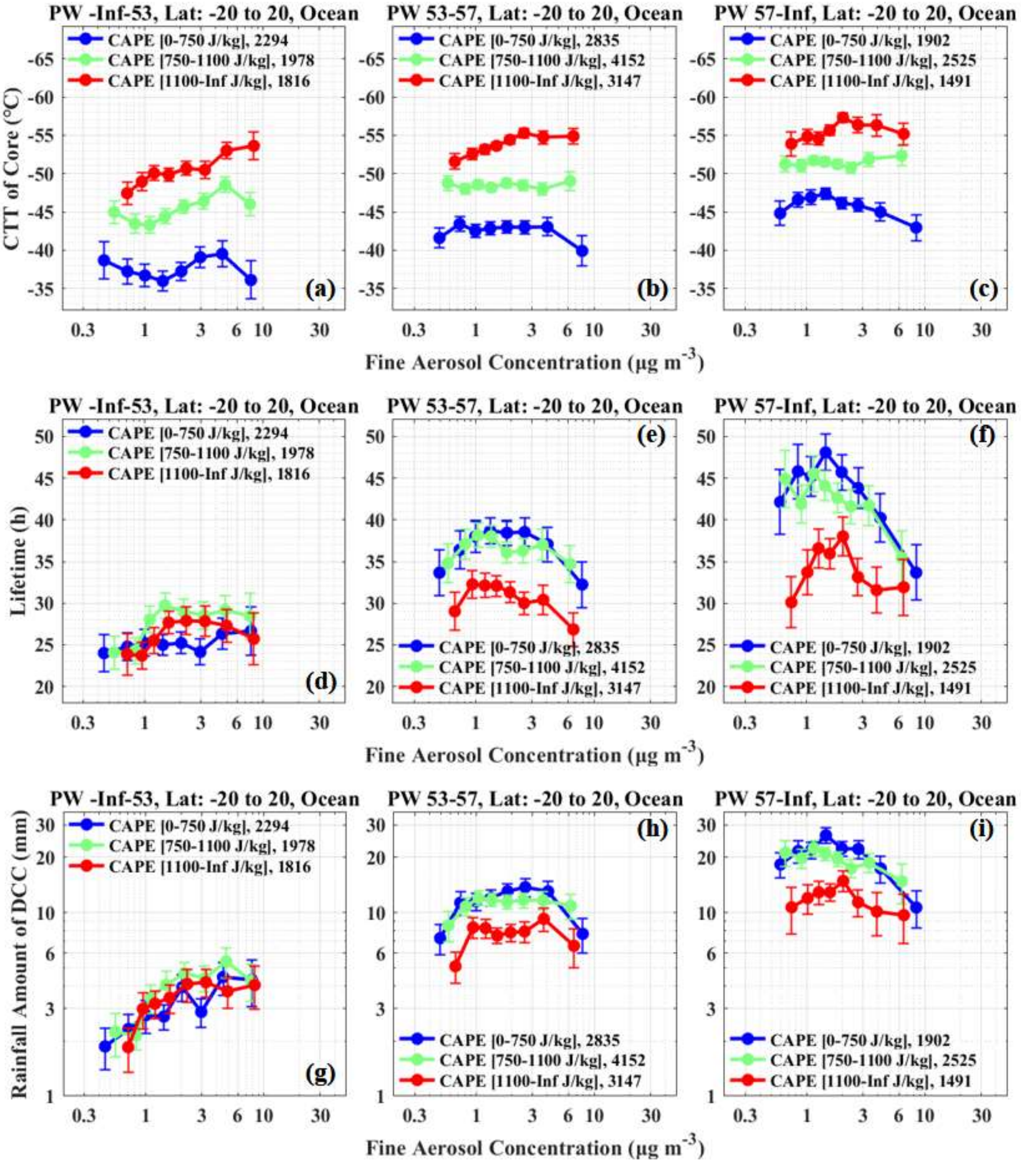

Supplementary Fig. 2 | Same as Supplementary Fig. 1, but over ocean.

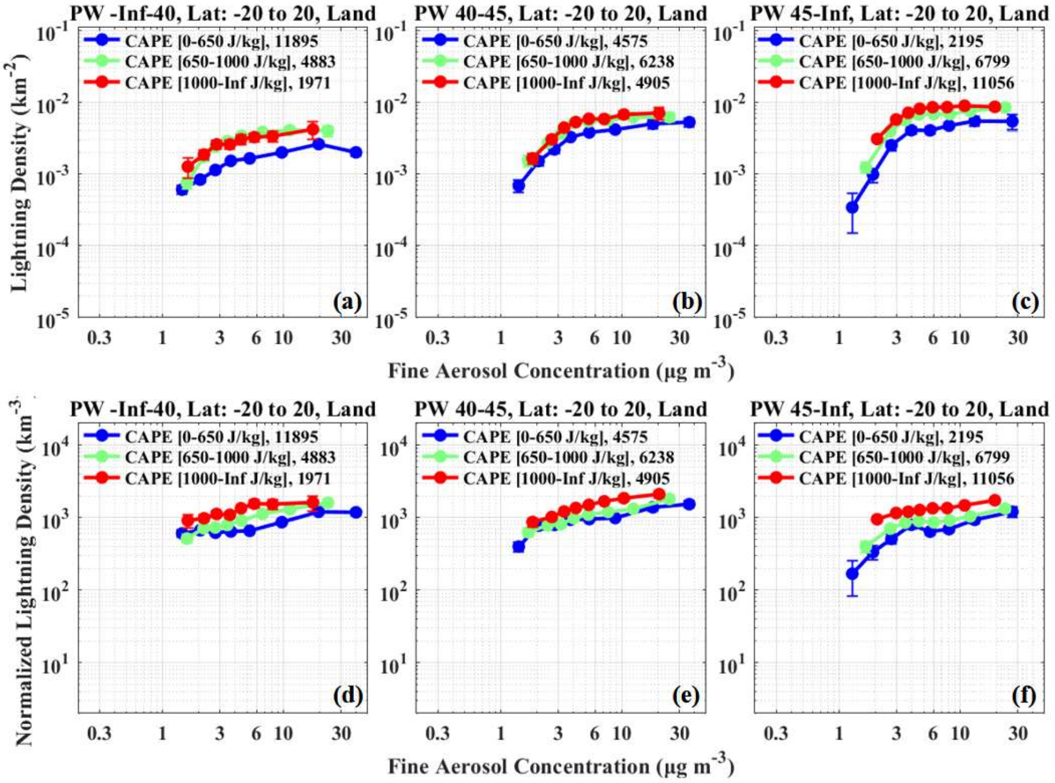

Supplementary Fig. 3 | Same as Supplementary Fig. 1, but for (a-c) lightning density and (d-f) normalized lightning density over land.

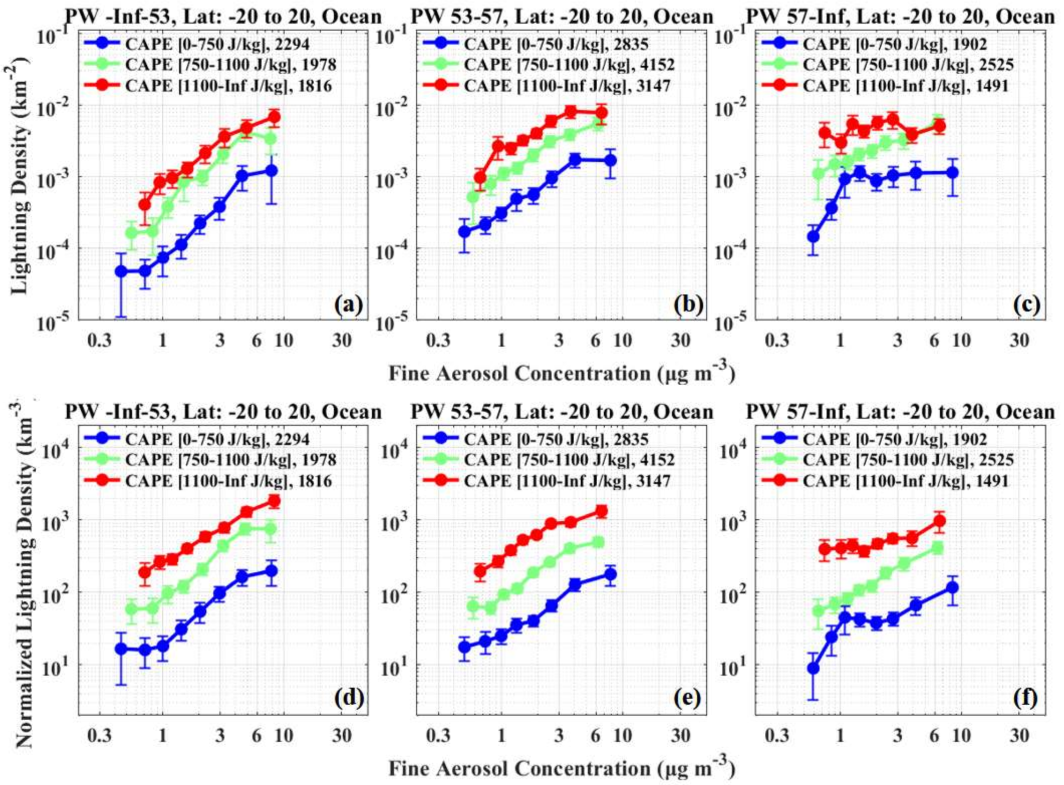

Supplementary Fig. 4 | Same as Supplementary Fig. 3, but over ocean.

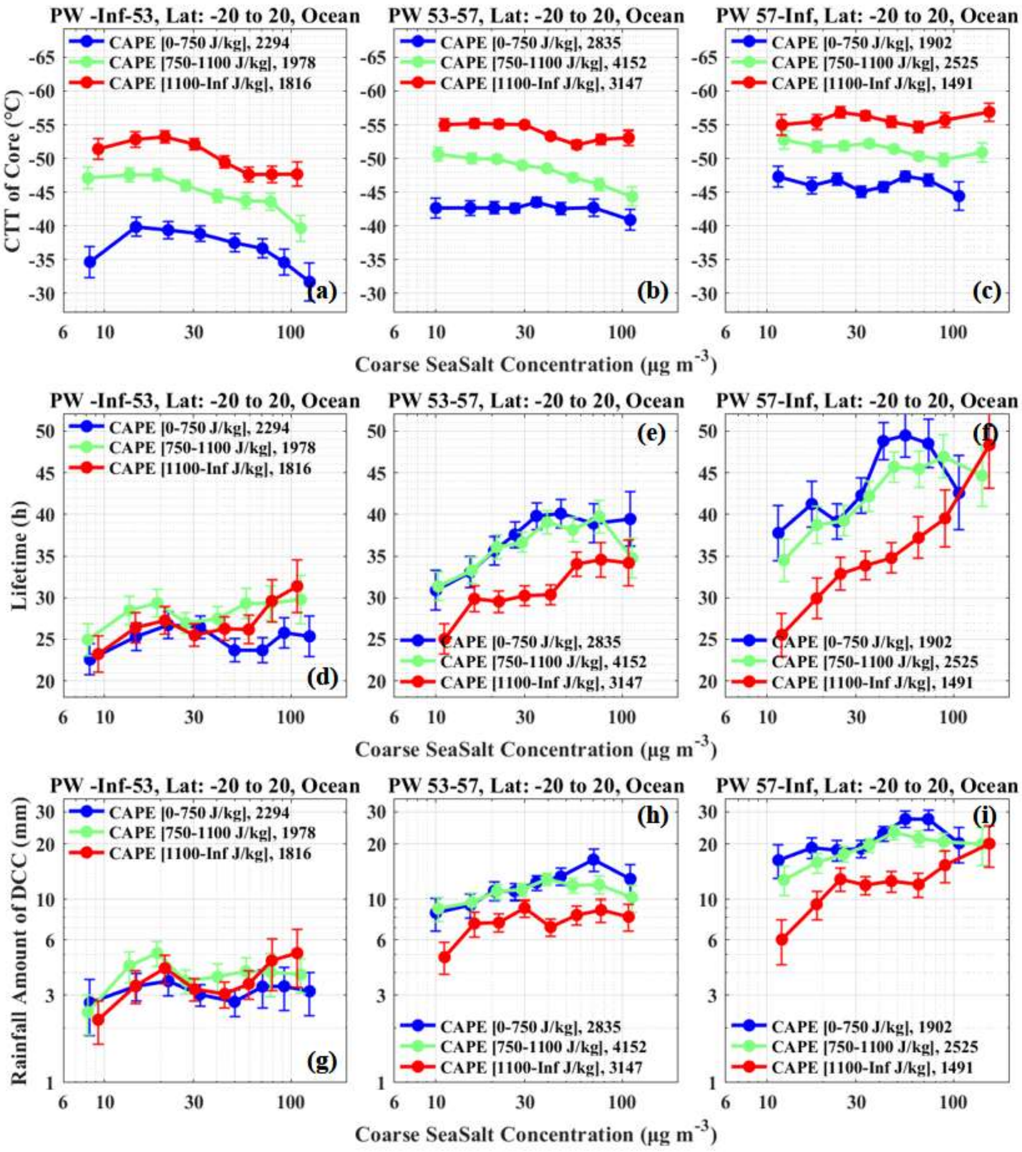

Supplementary Fig. 5 | Aerosol-driven deep concective cloud (DCC) variations with coarse sea salt aerosol at the ocean surface. (a-c) cloud top temperature (CTT) of core, (d-f) lifetime and (g-i) rainfall amount of DCC variations are showed under different fixed intervals of precipitable water (PW) over ocean, respectively. Three lines with different colors (blue, green and red) indicate the low, moderate and high convective available potential energy (CAPE). The I-type vertical bars indicate standard error. The same percentile of 5%, 15%, 30%, 50%, 70%, 85%, 95%, 100% for fine aerosol bins are used over ocean, respectively. The total number of data points for each line is shown in the legend.

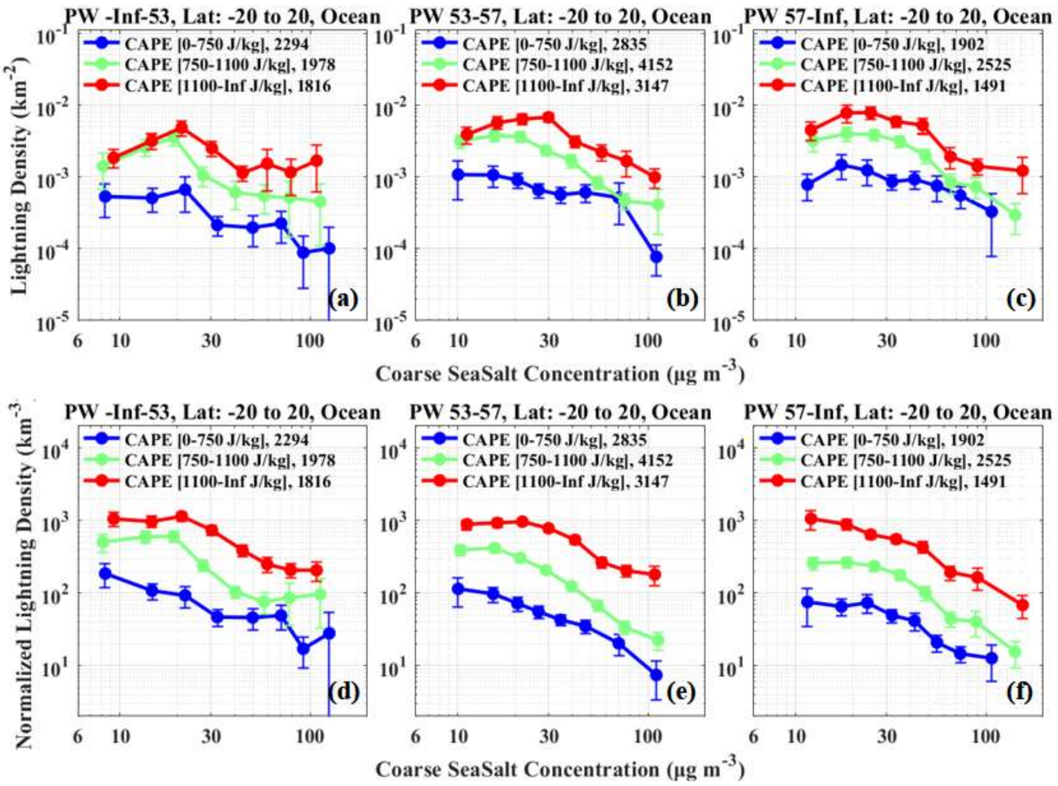

Supplementary Fig. 6 | Same as Supplementary Fig. 5, but for (a-c) lightning density and (d-f) normalized lightning density over ocean.

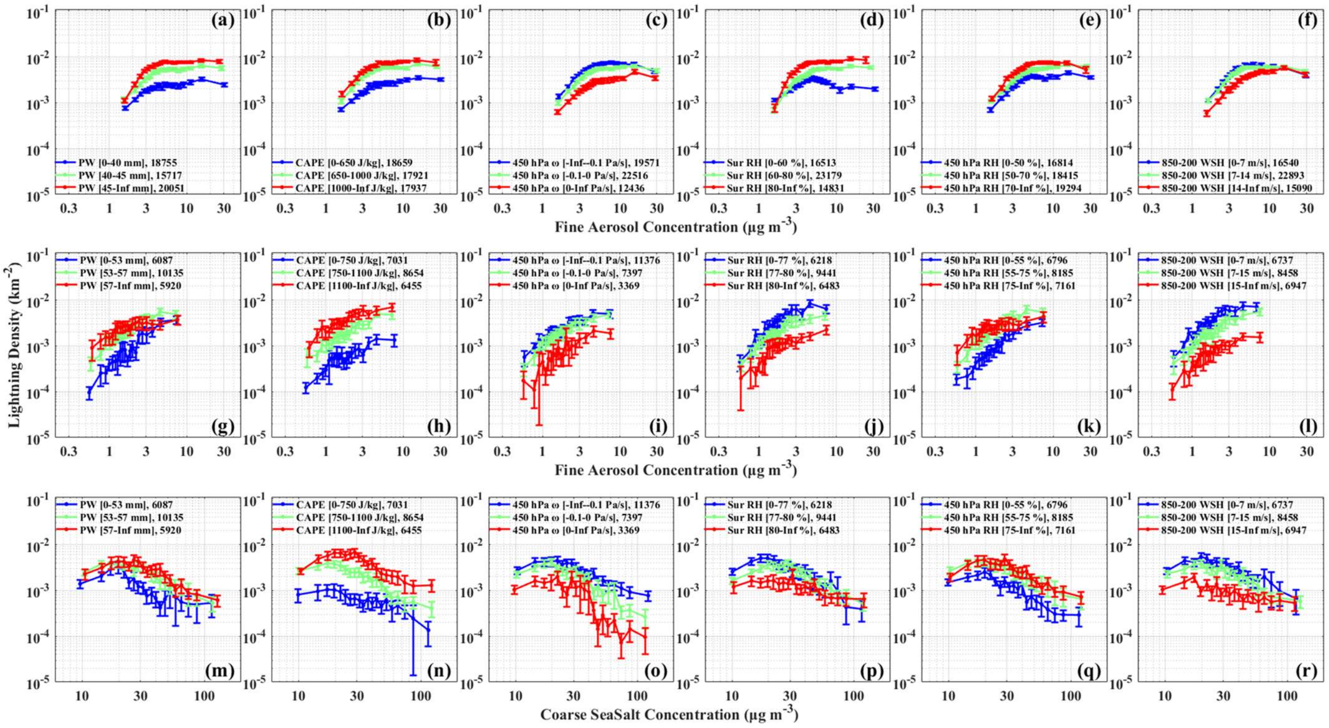

Supplementary Fig. 7 | Aerosol-driven lightning density variations at three classes of various meteorological parameters. The first two rows show the results for fine aerosol over land and ocean, respectively. The third row shows the results for coarse sea salt over ocean. The multiple meteorological parameters are discussed, including (a) precipitable water (PW), (b) convective available potential energy (CAPE), (c) 450 hPa ω, (d) surface relative humidity (RH), (e) 450 hPa RH, and (f) 850–200 hPa wind shear (WSH). The meteorology of (g-l) and (m-r) are the same as a-f, but with fine aerosol and coarse sea salt over ocean, respectively. The I-type vertical bars indicate standard error. The aerosol bins are at intervals of 5% of the cases.

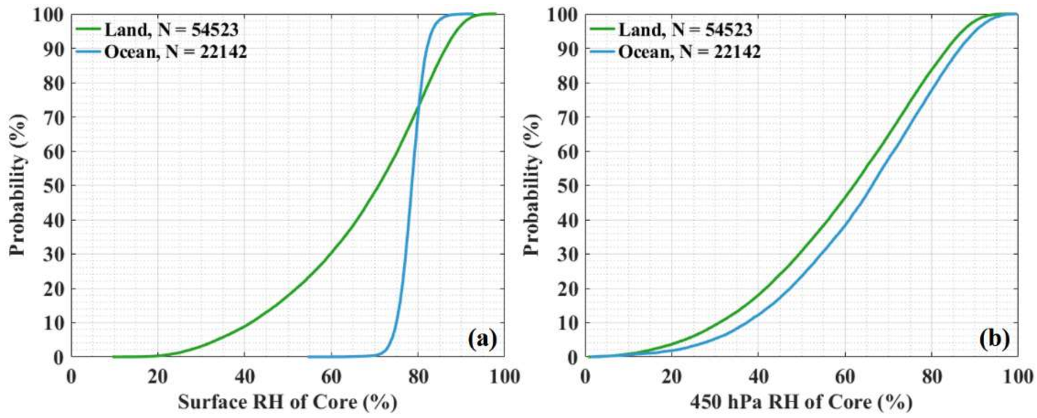

Supplementary Fig. 8 | Comparison of cumulative probability distribution about relative humidity (RH) on core area over land and ocean. The RH is obtained from the surface (left, a) and 450 hPa (right, b) levels, respectively, based on NCEP reanalysis data.

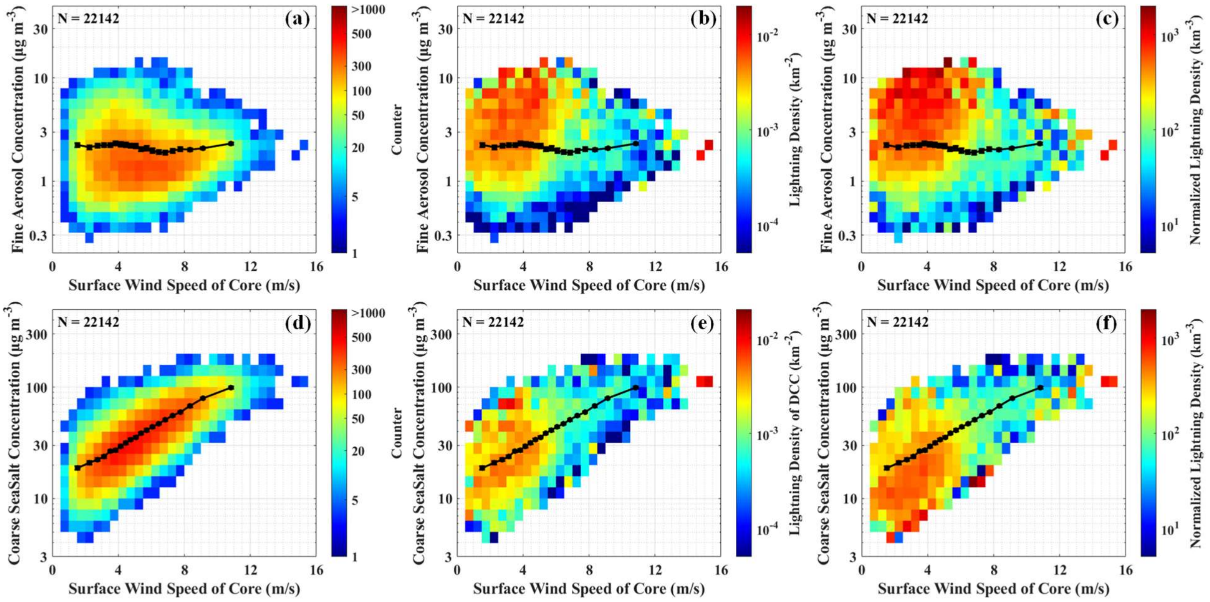

Supplementary Fig. 9 | Correlation among lightning, surface wind speed, and fine aerosol (top, a-c) and coarse sea salt (bottom, d-f) over ocean, respectively. Color indicates the simple size (left), lightning density (middle) and normalized lightning density (right) of deep concective cloud (DCC). The black dots indicate the average on x-axis bins at intervals of 5% of the cases. The I-type vertical bars indicate standard error. The total number of data points for each line is shown in the legend. The normalized lightning frequency for a captured DCC is defined as the total number throughout its lifetime at a fixed window of 10° × 5° per km^3 of integrated rainfall volume. It is equal to the lightning density divided by the rainfall amount of DCC.

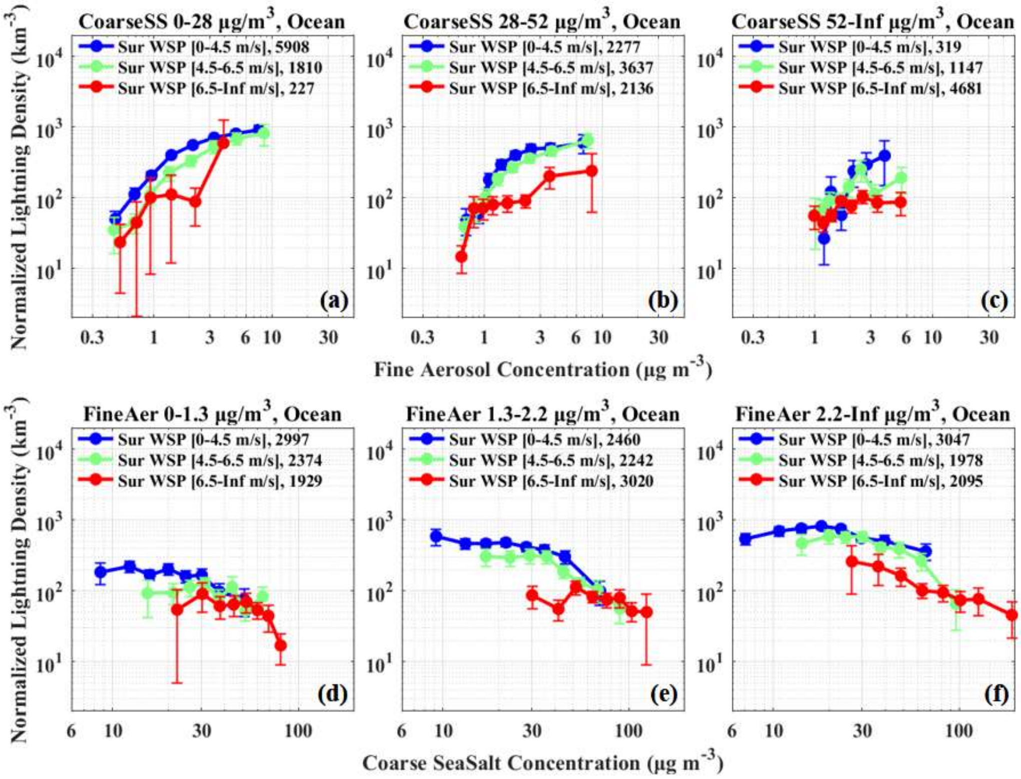

Supplementary Fig. 10 | Aerosol-driven variations of normalized lightning density with fine aerosol (top, a-c) and coarse sea salt (bottom, d-f) at surface over ocean, respectively. Three lines with different colors (blue, green and red) indicate the low, moderate and high surface wind speed (WSP) from NCEP reanalysis data. The I-type vertical bars indicate standard error. The percentile of 5%, 15%, 30%, 50%, 70%, 85%, 95%, 100% for aerosol bins are used over ocean. The total number of data points for each line is shown in the legend.

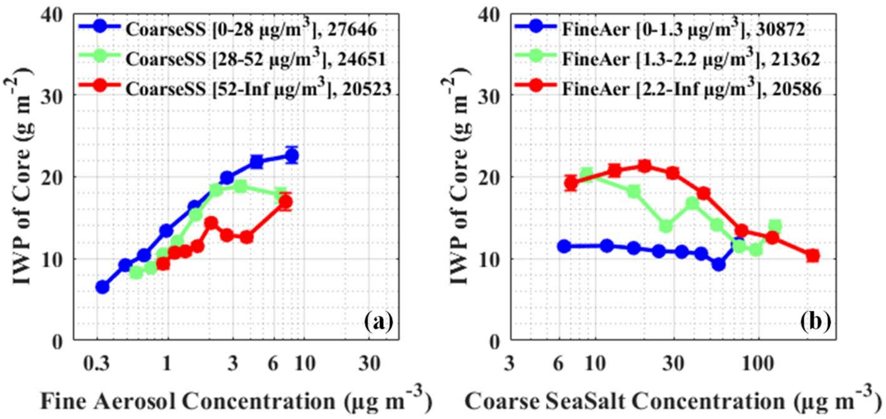

Supplementary Fig. 11 | Aerosol-driven variations of ice water path (IWP) at convective precipitation with fine aerosol (a) and coarse sea salt (b) at surface over ocean, respectively. Three lines with different colors (blue, green and red) indicate the low, moderate and high coarse sea salt or fine aerosol. The I-type vertical bars indicate standard error. The percentile of 5%, 15%, 30%, 50%, 70%, 85%, 95%, 100% for aerosol bins are used over ocean. The total number of data points for each line is shown in the legend. The IWP is calculated based on radar reflectivity factor from Global Precipitation Measurement (GPM) Dual-frequency Precipitation Radar observations in 2017, referring to the previous studies[10-12]. A convective precipitation is selected as its rainfall rate > 1 mm/h with a cloud with ice phase top, and is further identified by GPM 2ADPR product algorithm.

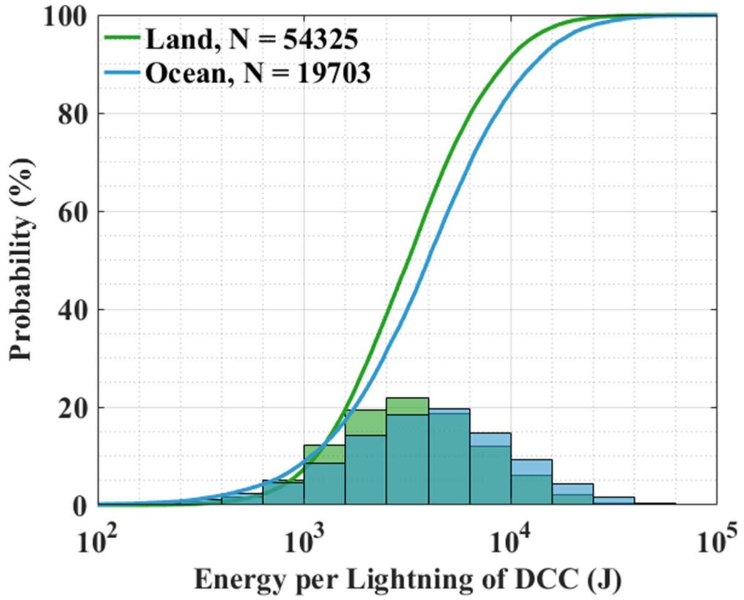

Supplementary Fig. 12 | Normalized probability density functions and corresponding cumulative distributions for the detected energy per lightning of tracked deep concective cloud (DCC) over land (green) and ocean (cyan) in this study, respectively. The lightning energy is the root-mean-square (RMS) electric field value of the triggered sferic waveform between 5 and 18 kHz with 1.3 ms waveform sampling [6, 13].

Supplementary Table 1 | Statistics of aerosol invigoration for deep concective cloud (DCC) properties and lightning at the 3×3 fixed precipitable water (PW) and convective available potential energy (CAPE). ΔCTT (cloud top temperature) indicates the change of CTT of core from clean to the optimal condition of fine aerosol over land and ocean. The number of lifetime, rainfall amount, lightning density and normalized lightning density indicate the enhancement factor from clean to the optimal condition of fine aerosol. The data was binned into three approximately equal size classes on PW and CAPE over land and ocean, respectively.

			ΔCTT (°C)	Factor of Lifetime (h)	Factor of Rainfall Amount (mm)	Factor of Lightning Density (kg^{-2})	Factor of Normalized Lightning Density (kg^{-3})
Land	Low PW	Low CAPE	-14.23	1.16	2.68	5.37	2.20
		Middle CAPE	-10.70	1.37	2.65	6.15	3.21
		High CAPE	-9.97	1.39	2.34	3.94	1.83
	Middle PW	Low CAPE	-16.03	1.21	2.44	9.17	4.34
		Middle CAPE	-10.18	1.20	1.96	4.34	2.88
		High CAPE	-6.64	1.29	2.19	4.89	2.53
	High PW	Low CAPE	-20.59	1.26	2.20	18.32	8.82
		Middle CAPE	-10.06	1.29	2.12	7.46	3.54
		High CAPE	-5.16	1.32	1.96	3.08	1.89
	Averaged		-11.51±4.49	1.28±0.07	2.28±0.25	6.97±4.38	3.47±2.04
Ocean	Low PW	Low CAPE	-0.84	1.11	2.39	25.44	11.94
		Middle CAPE	-3.58	1.23	2.43	25.72	12.97
		High CAPE	-6.19	1.17	2.26	16.74	9.75
	Middle PW	Low CAPE	-1.82	1.15	1.89	10.06	10.13
		Middle CAPE	-0.25	1.10	1.41	10.64	7.71
		High CAPE	-3.73	1.11	1.81	8.39	6.83
	High PW	Low CAPE	-2.58	1.14	1.44	7.83	13.04
		Middle CAPE	-1.10	1.01	1.06	5.17	7.51
		High CAPE	-3.43	1.26	1.39	1.54	2.46
	Averaged		-2.61±1.74	1.14±0.07	1.79±0.47	12.39±8.04	9.15±3.23

Supplementary References

1. Williams E, Stanfill S. The physical origin of the land–ocean contrast in lightning activity. *Comptes Rendus Physique* **3**, 1277-1292 (2002).
2. Pan Z*, et al.* Observational Quantification of Aerosol Invigoration for Deep Convective Cloud Lifecycle Properties Based on Geostationary Satellite. *Journal of Geophysical Research: Atmospheres* **126**, e2020JD034275 (2021).
3. Randles CA*, et al.* The MERRA-2 Aerosol Reanalysis, 1980 Onward. Part I: System Description and Data Assimilation Evaluation. *Journal of Climate* **30**, 6823-6850 (2017).
4. Rodger CJ*, et al.* Detection efficiency of the VLF World-Wide Lightning Location Network (WWLLN): initial case study. *Ann Geophys* **24**, 3197-3214 (2006).
5. Virts KS*, et al.* Highlights of a New Ground-Based, Hourly Global Lightning Climatology. *Bulletin of the American Meteorological Society* **94**, 1381-1391 (2013).
6. Hutchins ML*, et al.* Relative detection efficiency of the World Wide Lightning Location Network. *Radio Science* **47**, (2012).
7. Holzworth RH*, et al.* Global Distribution of Superbolts. *Journal of Geophysical Research: Atmospheres* **124**, 9996-10005 (2019).
8. Jacobson AR*, et al.* Performance Assessment of the World Wide Lightning Location Network (WWLLN), Using the Los Alamos Sferic Array (LASA) as Ground Truth. *Journal of Atmospheric and Oceanic Technology* **23**, 1082-1092 (2006).
9. Thornton JA*, et al.* Lightning enhancement over major oceanic shipping lanes. *Geophysical Research Letters* **44**, 9102-9111 (2017).
10. Petersen WA*, et al.* TRMM observations of the global relationship between ice water content and lightning. *Geophysical Research Letters* **32**, (2005).
11. Petersen WA, Rutledge SA. Regional Variability in Tropical Convection: Observations from TRMM. *Journal of Climate* **14**, 3566-3586 (2001).
12. Black RA. Radar Reflectivity-Ice Water Content Relationships for Use above the Melting Level in Hurricanes. *Journal of Applied Meteorology and Climatology* **29**, 955-961 (1990).
13. Hutchins ML*, et al.* Far-Field Power of Lightning Strokes as Measured by the World Wide Lightning Location Network. *Journal of Atmospheric and Oceanic Technology* **29**, 1102-1110 (2012).
